# An individual‐based model of seed‐ and rhizome‐propagated perennial plant species and sustainable management of *Sorghum halepense* in soybean production systems in Argentina

**DOI:** 10.1002/ece3.5578

**Published:** 2019-08-19

**Authors:** Chun Liu, Julio A. Scursoni, Raúl Moreno, Ian A. Zelaya, María Sol Muñoz, Shiv S. Kaundun

**Affiliations:** ^1^ Herbicide Bioscience Syngenta Ltd Bracknell UK; ^2^ Departamento de Producción Vegetal, Facultad de Agronomía Universidad de Buenos Aires Buenos Aires Argentina; ^3^ Product Biology Syngenta Argentina Buenos Aires Argentina; ^4^ Product Biology Syngenta Colombia Santa Fé de Bogotá Colombia

**Keywords:** ACCase‐inhibiting herbicides, evolution of herbicide resistance, glyphosate, Johnsongrass, no‐tillage, population models, Sorgo de Alepo, vegetative (asexual) propagation

## Abstract

Perennial plants which propagate through both seeds and rhizomes are common in agricultural and nonagricultural systems. Due to their multifaceted life cycle, few population models are available for studying such species. We constructed a novel individual‐based model to examine the effects of ecological, evolutionary, and anthropogenic factors on the population dynamics of perennial species. To exemplify the application of the model, we presented a case study of an important weed, *Sorghum halepense* (L.) Pers. (Johnsongrass), in soybean productions in Argentina. The model encompasses a full perennial weed life cycle with both sexual (seeds) and asexual (rhizomes) propagations. The evolution of herbicide resistance was modeled based on either single genes or quantitative effects. Field experiments were conducted in the species' native environment in Argentina to parameterize the model. Simulation results showed that resistance conferred by single‐gene mutations was predominantly affected by the initial frequency of resistance alleles and the associated fitness cost. Population dynamics were influenced by evolved resistance, soil tillage, and rhizome fecundity. Despite the pivotal role of rhizomes in driving the population dynamics of Johnsongrass, most herbicides target the aboveground biomass, and chemical solutions to control rhizomes are still very limited. To maintain effective (short‐term) and sustainable (long‐term) weed management, it is recommended to combine soil tillage with herbicide applications for suppressing the rhizomes and delaying the evolution of resistance. This novel model of seed‐ and rhizome‐propagated plants will also be a useful tool for studying the evolutionary processes of other perennial weeds, cash crops, and invasive species.

## 1 INTRODUCTION

Perennial plants which propagate through both seeds and rhizomes are commonly found in agricultural and nonagricultural systems. Perennial weed species, such as *Sorghum halepense* (L.) Pers. (Johnsongrass) in soybean production systems in Argentina, have become increasingly problematic and economically important (Binimelis, Pengue, & Monterroso, 2009). This is notably due to simplified tillage coupled with the prevalence of herbicide resistance (Nichols, Verhulst, Cox, & Govaerts, 2015). With the increasing evolution of resistance to glyphosate, use of acetyl‐CoA carboxylase (ACCase)‐inhibiting herbicides, such as haloxyfop and clethodim, have become the predominant chemical management option for Johnsongrass. Typically, Johnsongrass populations in Argentina are applied two to three times per season with haloxyfop or clethodim since 2010, imposing a high selection pressure for resistance. Some of the herbicide‐resistant traits are affected by a fitness penalty as assessed in *Alopecurus myosuroides* Hunds and *Lolium rigidum* Gaudin (Matzrafi, Gerson, Rubin, & Peleg, 2017; Menchari, Chauvel, Darmency, & Délye, 2008; Vila‐Aiub, Yu, Han, & Powles, 2015). Johnsongrass populations with multiple glyphosate and haloxyfop resistance have been documented in highly pressured regions such as Cordoba, Argentina, since 2015 (Heap, 2018). This brought challenges to agriculture in Argentina because there are not any other effective herbicide sites of action (SoAs) available on the market for controlling Johnsongrass. Failure to control Johnsongrass is a significant threat to soybean‐dominated agriculture in Argentina, since competition with crops can result in yield loss of ca. US$ 300 million a year (Beltrano & Montaldi, 1979; Colbert, 1979; Tuesca, Puricelli, Nisensohn, Faccini, & Papa, 1999). Considering current herbicide use and the widely adopted no‐tillage cropping systems, more cases of evolved herbicide resistance can be expected (Valverde, 2010), which if not addressed proactively or promptly will essentially mean growers have no herbicides to use in the near future.

Mathematical or population models are cost‐effective tools to study population dynamics and the evolution of herbicide resistance in important weed species (Renton, Busi, Neve, Thornby, & Vila‐Aiub, 2014). The effect of ecological, evolutionary, anthropogenic, and economic factors can be evaluated in these models, and accordingly weed management strategies can be optimized before they are recommended to growers. The common practice is to focus on a single dominant species, since the interactions between different weed species are difficult to quantify without sufficient data and incur additional uncertainty in model predictions. Past modeling efforts have focused primarily on annual weed species, such as *L. rigidum* (Monjardino, Pannell, & Powles, 2003), *A. myosuroides* (Colbach, Chauvel, Darmency, Délye, & Corre, 2016), and *Amaranthus* spp. (Lindsay et al., 2017; Liu et al., 2017; Neve, Norsworthy, Smith, & Zelaya, 2011). Perennial weed species, such as Johnsongrass, have seldom been modeled before, especially at the population level. The few models that are currently available either focus on describing the physiological relationship between temperature and seedling/bud emergence (e.g., Ghersa, Satorre, Esso, Pataro, & Elizagaray, 1990; Satorre, Ghersa, & Pataro, 1985) or make fuzzy logic assessment of herbicide resistance risk in different cropping systems based on anecdotal evidence without addressing biological or genetic mechanisms of evolution of resistance (Ferraro & Ghersa, 2013).

In the present study, we applied a mechanistic approach and present the first model that incorporates the creeping rhizomes (asexual propagation, aka vegetative propagation) as well as seeds (sexual propagation) for a complete life cycle of perennial plant species. The model is individual‐based, with each Johnsongrass plant having specific biological and genetic variables, parameterized on field experiments or literature data. The model was used to examine the effects of (a) ecological (i.e., fecundity, mortality, initial population density, emergence, and region), (b) evolutionary (i.e., frequency of resistance gene, fitness cost), and (c) anthropogenic (i.e., diversity in chemical program, soil tillage) factors on the population dynamics of Johnsongrass.

## 2 MATERIAL AND METHODS

### 2.1 Model design

The model description follows the ODD (Overview, Design concepts, Details) protocol for describing individual‐ and agent‐based models (Grimm et al., 2010). The model is implemented in NetLogo 6.0.4 (Wilensky, 1999). Simulations presented herein were parameterized specifically for Johnsongrass in Argentine soybean production systems. Importantly, the same model framework can be used in other perennial species if the species‐ and system‐specific data are available for parameterization.

#### 2.1.1 Purpose

The purpose of the model is to predict the response of Johnsongrass to chemical control and cultural practice at the population level, in terms of population density and propensity of herbicide resistance.

#### 2.1.2 Entities, state variables, and scales

Johnsongrass is a perennial Poaceae species with sexual reproduction via seeds. The species is monoecious and primarily self‐pollinated (autogamy); however, up to 5% cross‐pollination (allogamy) leads to genetic evolution and hybridization (Warwick & Black, 1983). In addition, Johnsongrass has vegetative propagation via rhizomes, which is an important venue for population dissemination and preservation.

**Figure 1:**
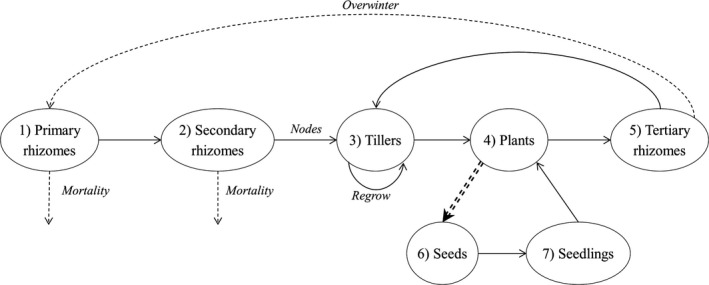
Life‐cycle diagram of Johnsongrass as implemented in the model. Solid arrows denote within‐season (i.e., crop season) life‐cycle processes, and dashed arrows denote between‐season processes. Single‐compound arrows denote asexual propagation, and double‐compound arrows denote sexual reproduction

In the model, seven life‐history stages were implemented as distinctive groups: seeds, seedlings, vegetative tillers, adult plants, and three tiers of rhizomes (primary, secondary, and tertiary) (Figure 1). Populations were modeled for 30 years to encompass enough life cycles to measure changes in population size and evolved herbicide resistance. Except for seeds (which are not modeled as individuals), each individual Johnsongrass has state variables characterizing its current resistance status—genotype (RR, RS, or SS) for target‐site resistance and phenotypic value *Pz* (the highest herbicide dose the plant can survive) for quantitative resistance. Seedlings and vegetative tillers are also characterized by their emergence date (Table 1, #13), which in turn determines which herbicide application(s) they are exposed to and number of seeds they produce (#9). Vegetative tillers have a state variable marking its apical position such that only the distal apical meristem from each crown of a rhizome is physiologically active (apical dominance; Beasley, 1970). If an apical tiller dies, a new tiller regrows from the distal meristem in the same rhizome. Additionally, rhizomes are characterized by the number of higher‐tier rhizomes and meristems they bear (#11 and #12).

**Table 1:** Parameters, values, and reference

Category	#	Parameter name	Value^a^ and unit	Reference and note	Varying values^b^ (figure)
Simulation	1	Density threshold	**5 plants/m^2^**	The model stops at densities above this level, and the weed control program is considered to have failed. In agricultural fields, Johnsongrass densities should be kept at lower level than this to ensure good crop yield	
	2	Number of replicates	**10,000**		
	3	Number of years	**30 years**		
	4	Field size	**10,000 m^2^**		
Ecological	5	Proportion of self‐pollination	**0.95**	Tarr (1962)	Fixed value
	6	Initial seedbank density	**10 seeds/m^2^**	In the beginning of the season in year 0	±10% (Figure 2)
	7	Initial rhizome density	**1 rhizome/m^2^**	In the beginning of the season in year 0	±10% (Figure 2)
	8	Proportion of seedling germination	**0.26**	As a result of seed predation and loss of viability. Egley and Chandler (1978); Looker (1981); Scopel et al. (1988); Van Esso and Ghersa (1989); Warwick and Black (1983)	±10% (Figure 2)
	9	Number of seeds produced per plant	Equation 2	Field experiment. Limited to be equal to or smaller than maximum values (#10)	
		North		Tartagal	(Figure 3)
		a_1_	1,554,053		
		b_1_	−0.066		
		South		Colón	±10% (Figure 2)
		a_2_	**298,660**		
		b_2_	**−0.066**		
	10	Maximum seeds produced per plant in the field			
		North	1,852 seeds/plant	Tartagal	(Figure 3)
		South	**356 seeds/plant**	Colón	±10% simultaneously with #9
	11	Average number of secondary rhizomes produced by per primary rhizome	**2**	Based on field observation. Implemented as a Poisson distribution	±10% (Figure 2)
	12	Average number of nodes on each secondary rhizome	**3**	Based on field observation. Implemented as a Poisson distribution	±10% (Figure 2)
	13	Emergence date	Equation 1	Field experiment. Two‐parameter Weibull distribution	
		Seedlings in the South		Colón	±1 day (Figure 2)
		Scale parameter *λ*	**169**		
		Shape parameter *k*	**10.5**		
		Seedlings in the North		Tartagal	(Figure 3)
		Scale parameter *λ*	165		
		Shape parameter *k*	4.1		
		Tillers in the South		Colón	±1 day (Figure 2)
		Scale parameter *λ*	**160**		
		Shape parameter *k*	**6.5**		
		Tillers in the North		Tartagal	(Figure 3)
		Scale parameter *λ*	165		
		Shape parameter *k*	5.2		
	14	Rhizome winter mortality			
		Tillage			(Figure 3)
		South	50%	Colón	
		North	40%	Tartagal	
		No‐tillage			
		South	**25%**	Colón	±10% (Figure 2)
		North	10%	Tartagal	(Figure 3)
Evolutionary		Glyphosate			
	15	Initial LD_50_	85, 139 and **1,719** g a.e./ha	a.e. = acid equivalents. Equivalent to 0.00002%, 0.002% and 80% resistant individuals, for comparison with ACCase‐R	(Figure 3)
	16	Phenotypic variance	**0.5087**	After Liu et al. (2017)	
	17	Ratio of average phenotype (*µ*) between offspring and parents	**1**	Tested in *a priori* simulations	Fixed value
	18	Ratio of phenotypic variation (*σ*) between offspring and parents	**1.18**	Tested in *a priori* simulations	Fixed value
		ACCase‐inhibiting herbicides			
	19	Initial frequency of alleles resistant to ACCase‐inhibiting herbicides	10^–7^ and **10^–5^**	Equivalent to 0.00002% and 0.002% resistant individuals	(Figure 3)
	20	Dominance of ACCase resistance gene	**1**	Kaundun (2014)	Fixed value
	21	Fitness cost (% reduction in survival or fecundity of ACCase‐resistant vs. ACCase‐sensitive individuals)			
		Literature	**42% for plant survival and 36% for seed production**	Menchari et al. (2008)	
		Max	90% for plant survival and seed production	Assumption	(Figure 3)
		None	No reduction in either survival or seed production	Assumption	(Figure 3)
Anthropogenic	22	Soybean sowing date			
		South	**16‐Dec**	138 days after the start of a season (DASS)	
		North	20‐Dec	142 DASS	(Figure 3)
		Application dates			
	23	Early POST		30 days after sowing	
		South	**15‐Jan**	168 DASS	
		North	19‐Jan	172 DASS	(Figure 3)
	24	Late POST		Assumed to cover all remaining plants in the field after early POST. Reduced efficacy represents both the lower control on large plants that escaped early POST and the missed control on plants emerging after late POST	
	25	Herbicide efficacy on aboveground plants	**95% for early POST and 90% for late POST**	Expert knowledge and field trial results	
	26	Herbicide efficacy on rhizomes			
		No‐tillage	**25%**		
		Tillage	50%		(Figure 3)
	27	Glyphosate application dose	**1,120 g a.e./ha**		

**Figure 2:**
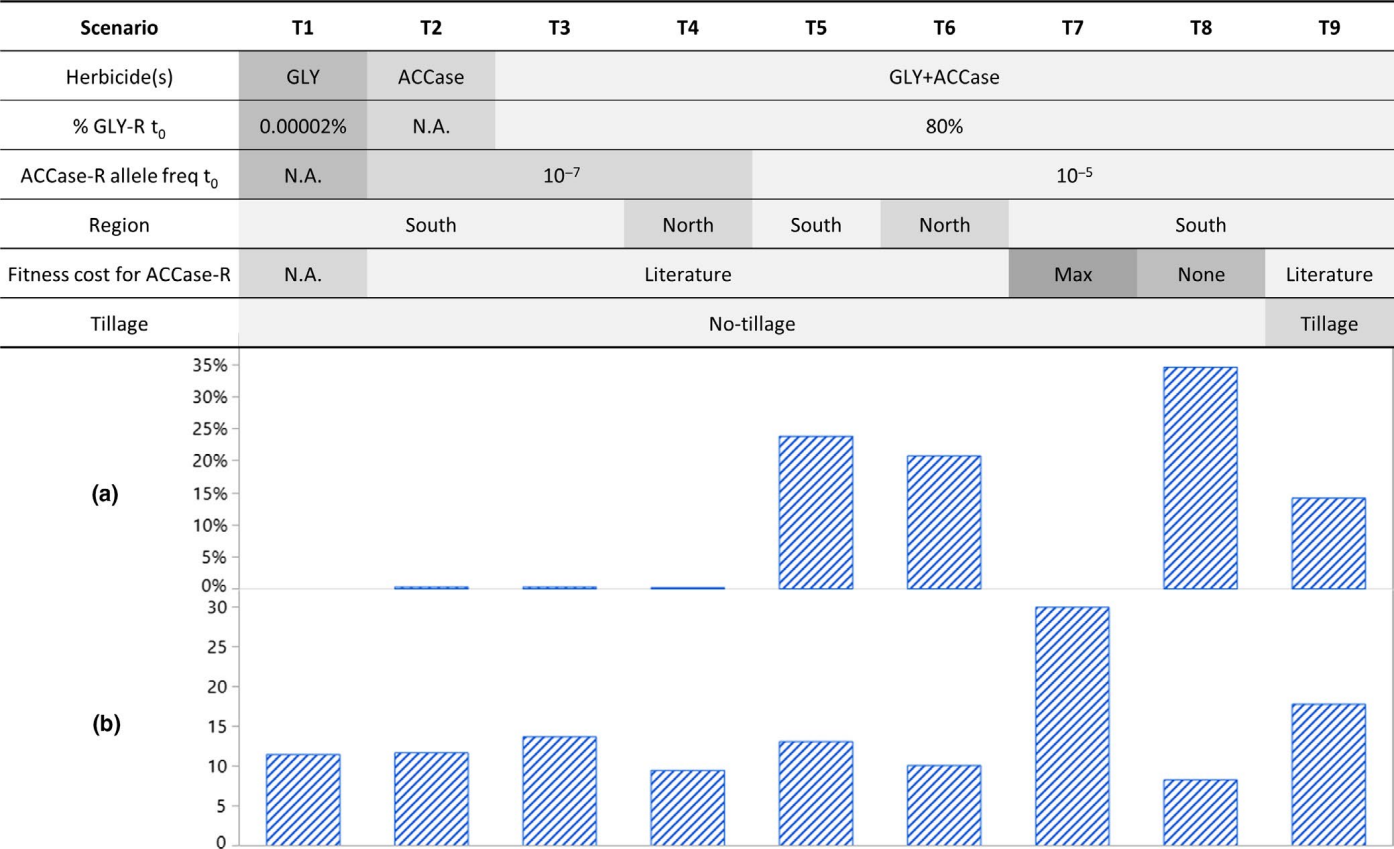
Predicted (a) ACCase‐R probability, and (b) failure year, with varying parameter and simulation settings. Scenario T5 was used as the baseline in the sensitivity analysis (Figure 3)

**Figure 3:**
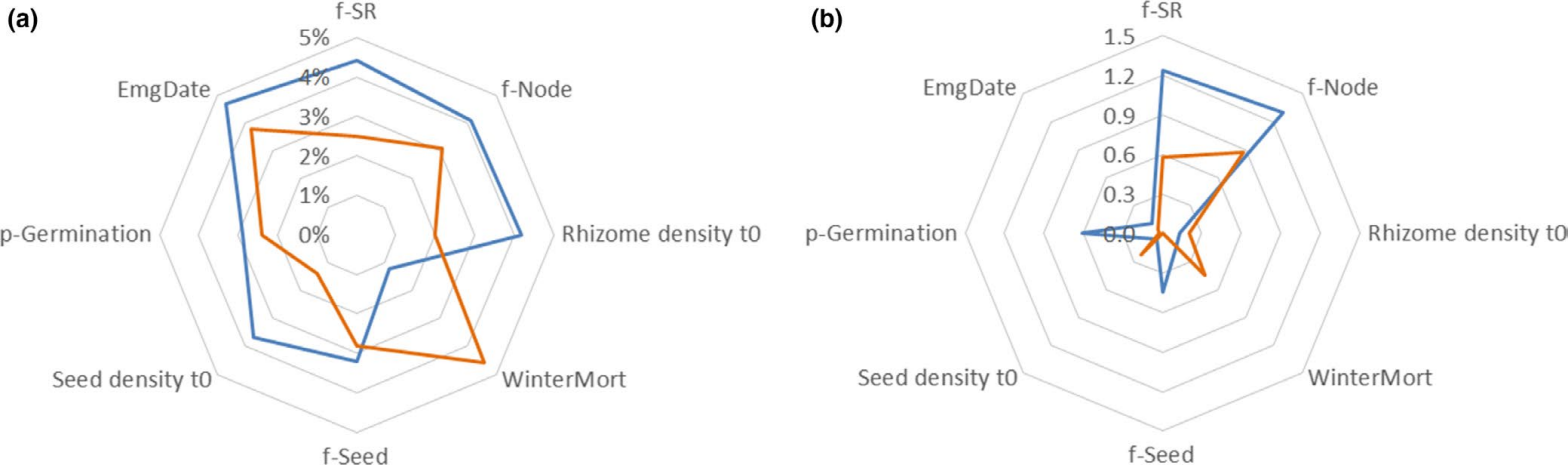
Changes in (a) ACCase‐R probability and (b) average failure year, after a 10% decrease (blue line) or increase (orange line) in seven ecological parameters: number of secondary rhizomes per primary rhizome (f‐SR), number of nodes per secondary rhizome (f‐Node), number of seeds per plant (f‐Seed), initial seed density (Seed density *t*_0_), initial rhizome density (Rhizome density *t*_0_), probability of seed germination (p‐Germination), and winter mortality (WinterMort), as well as a −1 day (blue) or +1 day (orange) change in the emergence date of seedlings and tillers (EmgDate)

Note

a Values in the baseline scenario T5 are in bold.

b In the sensitivity analysis (Figure 3) or discrete scenarios (Figure 2). Unless stated as fixed, all parameters can be adjusted by the model user.

The model simulates a 10,000‐m^2^ agricultural field by default. Johnsongrass propagate until a ceiling density of 5 plants/m^2^ is reached, beyond which the weed control program is considered to have failed. Each scenario was run with 1,000 replicates. One time step in the model corresponds to one year in the life cycle of Johnsongrass.

#### 2.1.3 Process overview and scheduling

Key processes in the model are schematically described in Figure 1 and detailed in section Submodels.

#### 2.1.4 Design concepts

##### Basic principles

The model simulates a complete life cycle of a perennial Poaceae species which propagates via both seeds and rhizomes. Herbicide resistance is inherited in a quantitative pattern for glyphosate and via a single dominant gene for ACCase‐inhibiting herbicides. Interspecific competition with crops and subsequent effect on seed production is implemented via a correlation with the emergence date of each plant (Table 1, #9). The ultimate purpose of weed control is to drive populations to low density levels in the field and maintain sustainable management. Therefore, intraspecific competition which is mostly triggered at extremely high population densities is beyond the scope of this paper.

##### Emergence

Weed density and resistance level, indicated by LD_50_ (the median resistance phenotypic value, g a.e./ha) of glyphosate and frequency of resistance allele(s) of ACCase‐inhibiting herbicides, emerge from the behavior and fate of each individual during herbicide selection, reproduction, and survival.

##### Stochasticity

Emergence time and initial *Pz* are defined by Weibull and log‐normal distributions, respectively. Values for each individual weed plant are randomly drawn from the distributions. Probabilities, for example, winter mortality rate and herbicide efficacy, are implemented at the individual level via Bernoulli trials.

##### Collectives

For quantitative resistance, the population is divided by the herbicide application rate into subgroups of sensitive individuals (i.e., *Pz* < application rate) and resistant individuals (i.e., *Pz* ≥ application rate); for target‐site resistance, the division is based on the genotype of each individual, SS = sensitive, RR and RS = resistant.

##### Observation

Key measures from the model are (a) the probability of evolved resistance to ACCase‐inhibiting herbicides (denoted as “ACCase‐R” from hereon) and (b) weed density, designated by the year of weed control failure when density exceeds 5 plants/m^2^ (denoted as “failure year” from hereon).

#### 2.1.5 Initialization

The baseline (default) scenario represents a population which consists of 80% and 0.002% resistant individuals to glyphosate and ACCase‐inhibiting herbicides, respectively (Table 1).

#### 2.1.6 Input data

All parameter values are predefined (Table 1), and the model does not use any external data files.

#### 2.1.7 Submodels

##### Seedling and vegetative tiller emergence

At each time step in the model, only 26% of the soil seedbank emerge as seedlings, as a result of seed predation and loss of viability (Table 1, #8).

The emergence pattern of seedlings and tillers was modeled as a two‐parameter Weibull distribution ranging between 0 and infinity, with unit of days after the start of a season (DASS), parameterized based on the field study described in section Field experiments for model parameterisation. The probability density function of the Weibull distribution is as follows,

(1) $$f\left(x\right)=\frac{k}{\lambda }{\left(\frac{x}{\lambda }\right)}^{k-1}e^{-{\left(x/\lambda \right)}^{k}}\;x\geq 0$$

where *f(x)* denotes the proportion of individuals in the population with an emergence date of *x*, and *k* and *λ* are the shape and scale parameters of the distribution (Table 1, #13). The regrowth of tillers are instigated mainly by herbicide applications; therefore, their emergence date is set to three weeks after the application date.

##### Chemical control and cultural practice

The model assumes clean field at planting; hence, seedlings that emerge before soybean sowing are removed from the population. Postemergence herbicide application(s) control seedlings, tillers, and regrown tillers. To represent the less efficient translocation of active ingredients to the rhizomes than the aboveground biomass, as well as an enhanced translocation in rhizomes that were cut into shorter parts by tillage, the model implements herbicide efficacies in descending order for aboveground biomass, rhizomes in tillage scenarios, and rhizomes in no‐tillage scenarios (Table 1, #25 and #26). Resistant individuals survive the applications while the majority of sensitive individuals are eliminated by the herbicides. For instance, if a herbicide has 95% efficacy, then 5% of the sensitive individuals escape the application, survive, and reproduce. Tillage does not kill Johnsongrass directly, but rather increases herbicide efficacy on rhizomes (as described above) and reduces rhizome overwinter survival (Table 1, #26 and #14).

##### Sexual reproduction

The number of seeds that a Johnsongrass plant produces is set to correlate with its emergence time, representing the effect of interspecific competition with crops.

(2) $$f_{i}=a\times e^{b\times x}$$

where *f_i_* denotes fecundity, *x* denotes the emergence date (in units of DASS) of the grass, and *a* and *b* are regression parameters (Table 1, #9; section Field experiments for model parameterisation).

##### Asexual propagation

In the beginning of a season in the model, the underground population consists of overwintered primary rhizomes which produce secondary rhizomes. Tillers sprout from the nodes on the secondary rhizomes. Tertiary rhizomes are produced by adult plants, which consist of mature vegetative tillers and seedlings. Primary and secondary rhizomes either die or lose physiological activity after reproduction and are therefore removed from the model at the end of a season. Both primary and secondary rhizomes can branch and produce multiple secondary rhizomes and tillers, respectively, numbers of which were implemented via Poisson distributions (Table 1, #11 and #12).

##### Inheritance of herbicide resistance

In this model, herbicide resistance is considered to be inherited either in a quantitative fashion or via a single dominant gene. The former case is associated with typical resistance mechanisms such as non‐target‐site resistance (metabolism and/or impaired translocation) or gene amplification, for example, resistance to glyphosate (Vila‐Aiub et al., 2012), and the latter case represents target‐site resistance, for example, resistance to clethodim and haloxyfop (Kaundun, 2014). When an offspring emerges, either from sexual or asexual propagation, it inherits the genetic material from the parent(s). If sexual propagation through meiosis occurs, the genetic material of the progeny is comprised of 50% origin from the female (mother) plant and 50% from a randomly selected male (father) plant; if asexual propagation is present, 100% of the genetic material in the progeny is maternally inherited (clone).

###### *(a)* *Quantitative resistance*

In nonrandom mating systems as such, the offspring's *Pz* cannot be calculated from the Breeder's equation (Falconer & Mackay, 1996) as implemented in Liu et al. (2017); hence, a separate individual‐based model was built to make *a prior* simulations of *Pz*. In this separate model, 20 genes from each parent and seed were modeled as individual entities, contributing a modicum of resistance to the final *Pz*. The implementation was based on the theory of the infinitesimal model (Bulmer, 1971; Liu et al., 2017; Thoday & Thompson, 1976). The ratio between the mean Ln(*Pz*) in the seeds and the parents was calculated, as well as the ratio between the standard deviations of Ln(*Pz*). These ratios (Table 1, #17 and #18) were then applied in the Johnsongrass model to calculate the mean and standard deviation of *Pz* in the new seeds.

###### *(b)* *Single‐gene resistance*

Upon exposure to the herbicide, individuals with RR and RS genotypes survive in the model and those with SS genotype are mostly controlled, with some escapes indicated by the efficacy value. For the self‐pollinating parents, new seeds inherit both alleles from the same parent, that is, if the parent has a genotype of A_1_A_2_, new seeds will have A_1_A_1_, A_1_A_2_, or A_2_A_2_ genotypes. For outcrossing, the father plant is randomly selected, and new seeds inherit one allele from each of the parents, that is, if female parent has a genotype of A_1_A_2_ and male parent has A_3_A_4_, new seeds will have A_1_A_3_, A_1_A_4_, A_2_A_3_, or A_2_A_4_ genotypes. Recombination was not considered in the model.

##### Overwintering

After the simulated winter mortality, the remaining tertiary rhizomes survive to the next season and become the primary rhizomes that eventually produce secondary rhizomes. Aboveground biomass do not survive winter.

##### Fitness cost

Effect of fitness cost in resistant individuals can be considered in the model, via a reduction in the number of seeds produced by a mature plant and survival of seedlings and tillers. Although the quantitative effect may vary from plant to plant, the effect in the model was parameterized based on a study in black‐grass with the aspartate 2078 to glycine mutation in ACCase (denoted as “Literature” in the scenarios). In addition, extremely high (denoted as “Max”) and low (denoted as “None”) fitness cost were also tested for comparison (Table 1, #21).

### 2.2 Sensitivity analysis and management scenarios

Sensitivity analysis was performed on the baseline scenario where key ecological parameter values were varied by ±10% or ±1 day for emergence time (Table 1), and population‐level responses were measured. The model then assessed discrete scenarios to investigate the role of evolutionary and anthropogenic factors in the population dynamics of Johnsongrass (mini table in Figure 2).

### 2.3 Field experiments for model parameterization

During 2013–2014 season, field studies were conducted to parameterize emergence and seed production in the model. Populations of Johnsongrass previously confirmed resistant to glyphosate in two soybean production fields in Argentina were studied: Colón (Rolling Pampas, Central Argentina; 33 53′S 61 06′W; denoted as “South” in the model) and Tartagal (Salta Province, North Argentina; 22 30′S 63 50′W; denoted as “North” in the model). Twenty experimental quadrats (0.25 m^2^ each) were randomly distributed in each field. Soybean was planted on December 16 in Colón and on December 20 in Tartagal. The number of emerging seedlings and vegetative tillers (arising from rhizomes) was recorded during five evaluations (January 6 and 28, February 19, March 15, April 12) in Colón and six evaluations (December 18, January 2 and 23, February 14, March 6, April 2) in Tartagal in the growing season. Data were used to generate emergence curves (i.e., proportion of emerged plants vs. DASS) for seedlings and vegetative tillers, respectively (Equation 1; Table 1, #13).

Johnsongrass seeds were collected from the Colón and Tartagal populations. The seeds were conditioned for germination and grown to maturity in a restricted field zone in the Facultad de Agronomía, Universidad de Buenos Aires (FAUBA), where irrigation, fertilization, and adversities were controlled. The emergence time of these plants was recorded. The number of emerged panicles was counted from each plant and correlated with the emergence time as an exponential regression:

(3) $$N_{p}=4098.5\times e^{-0.066x}$$

where *N*_p_ denotes the number of panicles per plant, *x* denotes the emergence time of the plant; goodness of fit for the regression *R* ^2^ = 0.6403. In addition, average number of panicles per plant was annotated from plants that grew naturally in the two experiment fields (Colón: 1.81 panicles/plant; Tartagal: 2.8 panicles/plant) and compared with the result obtained in FAUBA (4 panicles/plant). This comparison was conducted to correct for difference between FAUBA and field‐grown plants. Finally, average number of seeds per panicle was counted in the two fields (Colón: 291 seeds/panicle; Tartagal: 1,517 seeds/panicle) and combined with the adjusted regression mentioned above to obtain a correlation between number of seeds per plant and emergence time (Equation 2; Table 1, #9).

### 2.4 Statistical analysis

Model simulations differ with empirical experiments in the way that statistical significance can be increased by generating more replicates in the former. Visual comparison of model outputs from multiple perspectives across different scenarios proves to be a more comprehensive approach than statistic test of significance of simulation results (Grimm & Railsback, 2005). Therefore, each scenario in the model was run with 1,000 replicates and both weed density and resistance probability were presented as population‐level outputs.

## 3 RESULTS

### 3.1 Effects of biological and ecological factors

In the baseline scenario, the ACCase‐R probability was 24%, with average failure year being 13 (Figure 2, T5). Natural variation among 10 × 1,000 replicates with the same setting in the baseline scenario was up to 5.8% difference in ACCase‐R probability and 0.2 years difference in the failure year; therefore, any changes smaller than these values would be considered as insensitive. Among the eight tested parameters associated with initial population density, emergence, fecundity, and mortality, none was sensitive with regard to resistance evolution (Figure 3a). However, with regard to weed density, fecundity of rhizomes (f‐SR and f‐Node) was the most sensitive parameter (Figure 3b), indicating the dominant contribution of asexual propagation to the population dynamics of this perennial species.

Regional difference, as a combination of seed production, emergence pattern, and rhizome winter mortality, did not lead to significant difference in resistance evolution but the weed control failed at least three years earlier in the North than the South (Figure 2, T3 vs. T4, T5 vs. T6). The average ratio between densities of the seedbank and the tertiary rhizomes was 5.1 in the South (T3) and 11.9 in the North (T4). This was consistent with observations in the field where aboveground populations were more abundant in the North while underground populations were better developed in the South (J. A. Scursoni, field observation).

### 3.2 Effects of genetic and evolutionary factors

When applied as the solo chemical treatment, with equally rare proportion (0.00002%) of resistant individuals in the beginning of the simulations, glyphosate was more prone to evolved resistance (8%, T1) than ACCase‐inhibiting herbicides (0.3%, T2). However, the onset of ACCase‐R was in the fourth year, which was up to four years earlier than that of glyphosate.

In more than 99.9% of all simulations, the probability of weed control failure (when density exceeded 5 plants/m^2^) synchronized with the probability of evolved resistance (to glyphosate in T1, or to ACCase‐inhibiting herbicides in T2–T9).The initial frequency of resistance alleles played a key role in ACCase‐R. With naturally low occurrence of resistance alleles (10^–7^), in 99.7% of the replicates in T2 and T3, ACCase‐R did not evolve and weed density was kept at low levels for at least 30 years (Figure 2. Note the average year of control failure in Figure 2b was calculated from the 0.3% of the simulations with evolved ACCase‐R). However, an increased resistance allele frequency of 10^–5^, which represented a larger number of gene mutations that confer resistance to haloxyfop (*cf*. clethodim), or dispersed seeds or propagated rhizomes from neighboring resistant fields, increased the ACCase‐R probability by 80 times (T5).

In addition to allele frequency, fitness penalty had significant influence on both resistance evolution and population density: Without any fitness penalty, the onset of evolved resistance was as early as the fourth year, leading to a 35% ACCase‐R probability and control failure in year 8 (T8); when fitness cost was implemented based on literature data, the onset of evolved resistance was delayed for one year, leading to an 11% lower ACCase‐R probability, and failure year was delayed for five years (T5); with extremely strong assumption of 90% fitness penalty, ACCase‐R did not evolve and density was well controlled for at least 30 years in all the 1,000 replicates (T7).

### 3.3 Effects of anthropogenic factors

If the chemical treatments consisted of a solo herbicide, in the 8% (T1) and 0.3% (T2) cases with evolved resistance, weed control failed in <12 years. With a more diversified herbicide program, the control failure was delayed for at least two years, despite that the populations were already highly resistant to glyphosate (T3). When the initial ACCase‐R allele frequency was further increased to 10^–5^ (T5), the average failure year was still one year later than the programs with solo herbicides in T1 and T2.

More importantly, soil tillage had predominant effect on weed density and was beneficial to the mitigation of resistance. In the cases where density exceeded control threshold due to ACCase‐R, the scenario with soil tillage (T9) had control failure at least four years later than that without tillage practice (T3 and T5), regardless of initial allele frequency. This is primarily explained by the enhanced winter mortality and herbicide efficacy on segmented rhizomes. No‐tillage also meant that weed management relied solely on chemical control, which consequently resulted in 24% ACCase‐R probability in T5, while only 14% of the replicates chronicled ACCase‐R in T9.

## 4 DISCUSSION

The simulation results suggested that the evolution of resistance was mainly driven by evolutionary parameters, such as initial level of resistance and fitness cost, while the weed population density was predominantly affected by anthropogenic factors, such as diversity in herbicide programs and soil tillage (Figure 2). This indicates that despite the perseverance of its perennial life‐cycle strategy, Johnsongrass remains to be manageable with the right chemical and cultural practices. In Argentina, Johnsongrass is one of the most common weed species in agricultural ecosystems. Before the first reported case of resistance in 2005, glyphosate had been the dominant herbicide for managing Johnsongrass (Vitta, Tuesca, & Puricelli, 2004). In many places, the population size was significantly reduced, and this response was effectively reflected by the low probability of evolved resistance to glyphosate in the model simulations (Figure 2, T1). Indeed, herbicide treatments used to be more effective than sole mechanical control, for example, glyphosate was reported to increase crop yield by ca. 50% in comparison with mechanical control (Alvarez, Buzio, & Lopez, 1983). However, in recent years, with more cases of glyphosate resistance, Johnsongrass has had a revival and is now mainly controlled by ACCase‐inhibiting herbicides. Seven different gene mutations have been found to confer resistance to ACCase‐inhibiting herbicides (Beckie & Tardif, 2012; Kaundun, 2014), all of which affect haloxyfop while only two of these mutations endow resistance to clethodim. To keep the model design simple for the purpose of demonstrating principles, we assumed one general gene, and the difference in number of resistance codons was inferred by a different initial frequency of resistance allele. For example, with a higher resistance allele frequency, haloxyfop is more prone to evolve resistance than clethodim. This is concurrent with field observations in, for example, Cordoba Province whereby populations were resistant to glyphosate and haloxyfop but sensitive to clethodim (Heap, 2018; Scursoni, Vera, Oreja, Kruk, & Fuente, 2019). Similar conclusions with regard to the importance of allele frequency were reached by Jasieniuk, Brûlé‐Babel, and Morrison (1996) and Gressel and Segal (1990). Future modeling efforts could benefit from theoretical advances in the statistical approximation of evolution of allele frequency and dynamics of polygenic traits, for example, Barton and Vladar (2009).

Due to significant soil erosion, lack of water, high cost of irrigation, huge scale of farmland, and a strong cultural resistance to tillage in the grower community in Argentina, no‐tillage has become the preferred practice in current agricultural systems. To date, more than 79% of the farmlands are no‐tillage fields (Peiretti & Dumanski, 2014), and more than 55% of the farmlands are with soybean monoculture. Despite the advantages of no‐tillage, such as better soil and water conservation and less disturbance to soil ecosystem services provided by, for example, earthworms (Peiretti & Dumanski, 2014), the negative effect of this practice on Johnsongrass control is also evident. Because the plants are taller with larger rhizomes than in conventional systems, herbicides are applied in sub‐optimal conditions. In tillage systems with thorough plowing, soil turnover rate is 100%, and ca. 50% of the rhizomes are left on the soil surface and thus killed by frost in winter. In addition, since rhizomes are cut into smaller segments by tillage, the efficacy of herbicides on these rhizomes and their tillers is higher. This is especially beneficial for the control of Johnsongrass populations that propagate almost exclusively via rhizomes. The simulation results suggested that soil tillage played an overriding role in supressing and maintaining Johnsongrass densities at manageable levels. In agreement with the experiments by Elverdin, Bedmar, and Leonardi (1989), our simulations suggested that cultivation in combination with herbicides was the most effective means of Johnsongrass management. Nevertheless, the ultimate agricultural practice should aim to maximize the overall agricultural sustainability, balancing between the risk of soil erosion caused by conventional tillage and the difficulty of weed control reinforced by zero tillage. In this regard, intermittent tillage, for example, once every two or three years, and disk plowing are being revisited. With disk plowing, soil turnover rate is only half of conventional tillage. Rhizomes are not lifted up to the soil surface, but cut into small pieces, where apical growth is encouraged and germination from rhizomes are more synchronized, making spray easier and more targeted. More importantly, active ingredients can reach the distal areas of small rhizomes more easily and control the rhizomes more effectively. In the past, herbicide application plus disk plowing which helps incorporating the chemicals into 10–12 cm has provided good control of Johnsongrass (Mitidieri, 1974).

The evolution of resistance is a complex process which involves not only the selection pressure imposed by herbicides, but also the population dynamics of the weed species in question. For Johnsongrass, propagation occurs mainly via rhizome growth instead of seed production (Figure 3). Scopel, Ballare, and Ghersa (1988) claimed that Johnsongrass populations could not be maintained by seeds alone; when seed bank loss is considered, the finite rate of increase (*λ*) is <1. Genetics of the rhizomes remained largely unchanged due to cloning; therefore, it is possible that most plants in a field are from the same origin, thus being all sensitive or all resistant, the latter implying that the whole field will be overtaken by resistant plants. The fast vegetative propagation may be analogous to fungicide resistance, where the asexual reproduction in haploid fungi results in mutations conferring resistance being immediately expressed and then directly exposed to selection. In contrast, for species such as *Digitaria insularis* (L.) Fedde, while leading a perennial life cycle, the majority of propagation is via seed production (Pyon, 1975), and so control should focus more on the aboveground vegetation. Factors other than population dynamics and herbicides include, for example, climate change. Leguizamón and Acciaresi (2014) predicted that an increase in temperature by 15% would result in 50% increase in relative growth rate of Johnsongrass within a 20‐ to 29‐day period.

The novel model presented here is an effort to study the interactions of the various factors involved in the process of evolution under anthropogenic selection pressure. While in this paper, we focused on the management of Johnsongrass in Argentine soybean fields, the application of the model is not restricted to a particular species or herbicide resistance. The structure of the model can be used to study other key perennial weeds, such as *D. insularis*, which is currently the most problematic weed species in soybean production in Brazil. Furthermore, the model can be adapted to represent other perennial cash crops or invasive plants which propagate via stolons (runners), tubers, bulbs, and woody crowns. It also provides a potential starting point for investigating energy allocation under extreme environmental conditions, to increase efficiency of nitrogen fixation in perennial plants, as well as to breed high‐yield perennial crops. More broadly, the theory and experiments of evolutionary rescue indicated that populations can potentially survive and recover from rapid environmental changes without relying on immigration (genetic rescue), provided that the population size is sufficiently large and genetic variability is sufficiently high (Bell & Gonzalez, 2009; Gonzalez, Ronce, Ferriere, & Hochberg Michael, 2013). However, these theoretical possibilities have only been proved by experiments in model systems, such as yeast (Gomulkiewicz & Shaw, 2013; Gonzalez et al., 2013). Weed populations provide a more realistic study system for evolutionary rescue; however, the selection pressure is recurrent meanwhile not strong enough, therefore, it is very rare that a weed population would be extirpated due to herbicide use. With modifications to adapt for stochastic extinction and emerging mutant, our model can be used to identify the boundary between evolutionary rescue theory and ecological realism.

## CONFLICT OF INTEREST

None declared.

## AUTHORS CONTRIBUTION

SSK and IAZ conceived the ideas; CL, JAS, RM, and IAZ designed the methodology; JAS and MSM conducted the field experiments, collected, and analyzed the field data; CL constructed the model, ran simulation experiments, analyzed the results, and led the writing of the manuscript. All authors contributed critically to the drafts and gave final approval for publication.

## Data Availability

Values of the model parameters are presented in Table 1, and simulation results can be downloaded from Dryad https://doi.org/10.5061/dryad.5km40nd.
